# Two precision medicine predictive tools for six malignant solid tumors: from gene-based research to clinical application

**DOI:** 10.1186/s12967-019-02151-8

**Published:** 2019-12-03

**Authors:** Zhiqiao Zhang, Tingshan He, Liwen Huang, Yanling Ouyang, Jing Li, Yiyan Huang, Peng Wang, Jianqiang Ding

**Affiliations:** Department of Infectious Diseases, Shunde Hospital, Southern Medical University, No. 1 Jiazi Road, Lunjiao, Shunde District, Foshan, 528308 Guangdong Province China

**Keywords:** Competitive endogenous RNA, Colorectal cancer, mRNA, Overall survival, Prognostic model

## Abstract

**Background:**

The current study aimed to construct competing endogenous RNA (ceRNA) regulation network and develop two precision medicine predictive tools for colorectal cancer (CRC).

**Methods:**

Differentially expressed (DE) analyses were performed between CRC tissues and normal tissues. A ceRNA regulation network was constructed based on DElncRNAs, DEmiRNAs, and DEmRNAs.

**Results:**

Fifteen mRNAs (ENDOU, MFN2, FASLG, SHOC2, VEGFA, ZFPM2, HOXC6, KLK10, DDIT4, LPGAT1, BEX4, DENND5B, PHF20L1, HSP90B1, and PSPC1) were identified as prognostic biomarkers for CRC by multivariate Cox regression. Then a Fifteen-mRNA signature was developed to predict overall survival for CRC patients. Concordance indexes were 0.817, 0.838, and 0.825 for 1-, 2- and 3-year overall survival. Patients with high risk scores have worse OS compared with patients with low risk scores.

**Conclusion:**

The current study provided deeper understanding of prognosis-related ceRNA regulatory network for CRC. Two precision medicine predictive tools named Smart Cancer Survival Predictive System and Gene Survival Analysis Screen System were constructed for CRC. These two precision medicine predictive tools can provide valuable precious individual mortality risk prediction before surgery and improve the individualized treatment decision-making.

## Introduction

Colorectal cancer (CRC) is one of the most prevalent malignant tumors worldwide. There were around 1.1 million newly diagnosed CRC patients and 0.55 million CRC patients died in 2018 [1]. The 5-year overall survival (OS) of CRC patients was less than 12% [2, 3]. Due to the poor overall survival, early detection of CRC patients with high mortality risk has important significance for improving the individualized treatment decision-making. Several prognostic models have been developed for CRC patients [4, 5]. However, the computational formulas of these prognostic models were too complex for clinical application by patients without calculation tools and medical knowledge. Additional, these prognostic models provided overall survival prediction for different groups, but not individual mortality risk prediction.

From the perspective of precise medicine, a good prognostic model should be able to provide individual mortality risk prediction for specific patient at the individual level. Considering the clinical need of precious individual mortality risk prediction for patients with different tumors, our research team has developed several precision medicine predictive tools for gastric cancer [6] and hepatocellular carcinoma [7]. For individual mortality risk prediction, our precision medicine predictive tools have the following advantages: full-time individual risk prediction, visual illustration, numerical presentation, customizable subgroups, and on-line computing.

So far the molecular biological regulatory mechanism of development and prognosis of tumor remains unclear. Several researches have explored underlying molecular biological regulatory mechanism for different tumors [8–11]. Salmena and collaborators presented an interesting molecular regulatory mechanism named competing endogenous RNAs (ceRNAs) [12]. The lncRNAs can indirectly regulate the expression of mRNAs through binding shared miRNA response elements [13]. Several researches have explored potential molecular regulatory mechanism for CRC [14–16]. Therefore the current research aimed to depict prognosis related ceRNA regulatory network and develop two precision medicine predictive tools for CRC patients.

## Materials and methods

### Study cohort

RNA sequencing and miRNA sequencing data were obtained from TCGA database. RNA sequencing data contained 14,449 lncRNAs and 20,337 mRNAs whereas miRNA sequencing data contained 1881 miRNAs. Four hundred and twenty-eight CRC patients were included after removing patients without complete survival information. GSE17538 dataset were downloaded from GEO database. GSE17538 dataset involved 231 CRC patients and 23,328 RNAs (GPL570 platform). The original read count values in TCGA dataset were normalized by log2 transformation. The gene background file (Gencode.v29 supplied by The European Bioinformatics Institute of The European Molecular Biology Laboratory (EMBL-EBI) database) was used for gene symbol annotation.

### Differentially expressed analyses and regulatory network

The original RNA data were processed by “edgeR” package, with a defined *P* of 0.05 and a ratio of 1.5 times between tumor and non-tumor tissues [17]. The original miRNA sequencing data were processed by “limma” package [18]. First, the interaction associations between lncRNAs and miRNAs were identified in miRcode database [19]. Second, miRTarBase [20], miRDB [21], and TargetScan [22] were searched for miRNA-targeted mRNAs. The ceRNA network was visualized by Cytoscape v3.6.1 [23].

### Statistical analyses and artificial intelligence algorithms

Random survival forest, Multi-task logistic regression, and Cox survival regression algorithms were carried out according to the algorithms suggested in the original articles [24–29]. Statistical analyses were carried out through SPSS Statistics 19.0 (SPSS Inc.,USA). Other analyses were carried out by R version 3.5.2 with corresponding packages. *P* value < 0.05 was defined as statistically significant.

## Results

### Baseline characteristics

TCGA dataset contained 428 CRC patients and GSE17538 dataset contained 231 CRC patients. The clinical information of included patients was shown in Table 1. The mortality of GSE17538 dataset was 40.3% (93/231), which was significantly higher than 22.9% (98/428) of TCGA dataset. There were significant differences in terms of survival time and pathological stage, whereas there were no significant differences in terms of age and gender.

**Table 1:** Clinical features of colorectal cancer patients

	TCGA cohort	GSE17538	*P*-value
Number [n]	428	231	NA
Death [n(%)]	98 (22.9)	93 (40.3)	< 0.001
Total survival time (mean ± SD, month)	29.8 ± 25.6	47.6 ± 30.6	< 0.001
Survival time for dead patients (month)	23.3 ± 22.7	26.8 ± 22.0	< 0.001
Survival time for living patients (month)	31.7 ± 26.1	61.7 ± 27.5	< 0.001
Age (mean ± SD, year)	66.5 ± 13.0	64.8 ± 13.4	0.100
Male [(n)%]	230 (53.7)	121 (52.4)	0.739
AJCC stage (IV/III/II/I/NA)	60/124/163/70/11	56/75/72/28/0	< 0.001
AJCC PT (T1/T0/NA)	51/294/72/11/0	NA	NA
AJCC PN (N2/N1/N0/NA)	76/103/249/0	NA	NA
AJCC PM (M2/M1/M0/NA)	47/60/315/6	NA	NA
Lymphovascular invasion (yes/no/NA)	148/237/43	NA	NA
Vascular invasion (yes/no/NA)	89/281/58	NA	NA
Residual tumor (3/2/1/0/NA)	23/21/4/307/73	NA	NA
Perineural invasion (yes/no/NA)	45/126/257	NA	NA
Grade (1/0/NA)	NA	55/144/32	NA

*SD* standard deviation, *NA* missing data, *AJCC* American Joint Committee on Cancer

### Differentially expressed analyses

Differential expression analysis could identify genes with significant differences in expression levels between normal samples and tumor samples. Compared with normal tissues, 3005 lncRNAs (2224 up-regulated and 781 down-regulated), 332 miRNAs (246 up-regulated and 86 down-regulated), and 6713 mRNAs (4087 up-regulated and 2626 down-regulated) were identified in CRC tissues. The volcano plots for differentially expressed RNAs were shown in Additional file 1: Figure S1.

### Screening of prognostic mRNAs

Univariate Cox regression was used to explore potential prognostic biomarkers for CRC. Out of previous differentially expressed mRNAs, there were 2504 mRNAs identified as prognostic biomarkers for CRC. Out of 2504 potential prognostic biomarkers, there were 1371 risk factors and 1133 protective factors.

### Development of ceRNA network

**Fig. 1:**
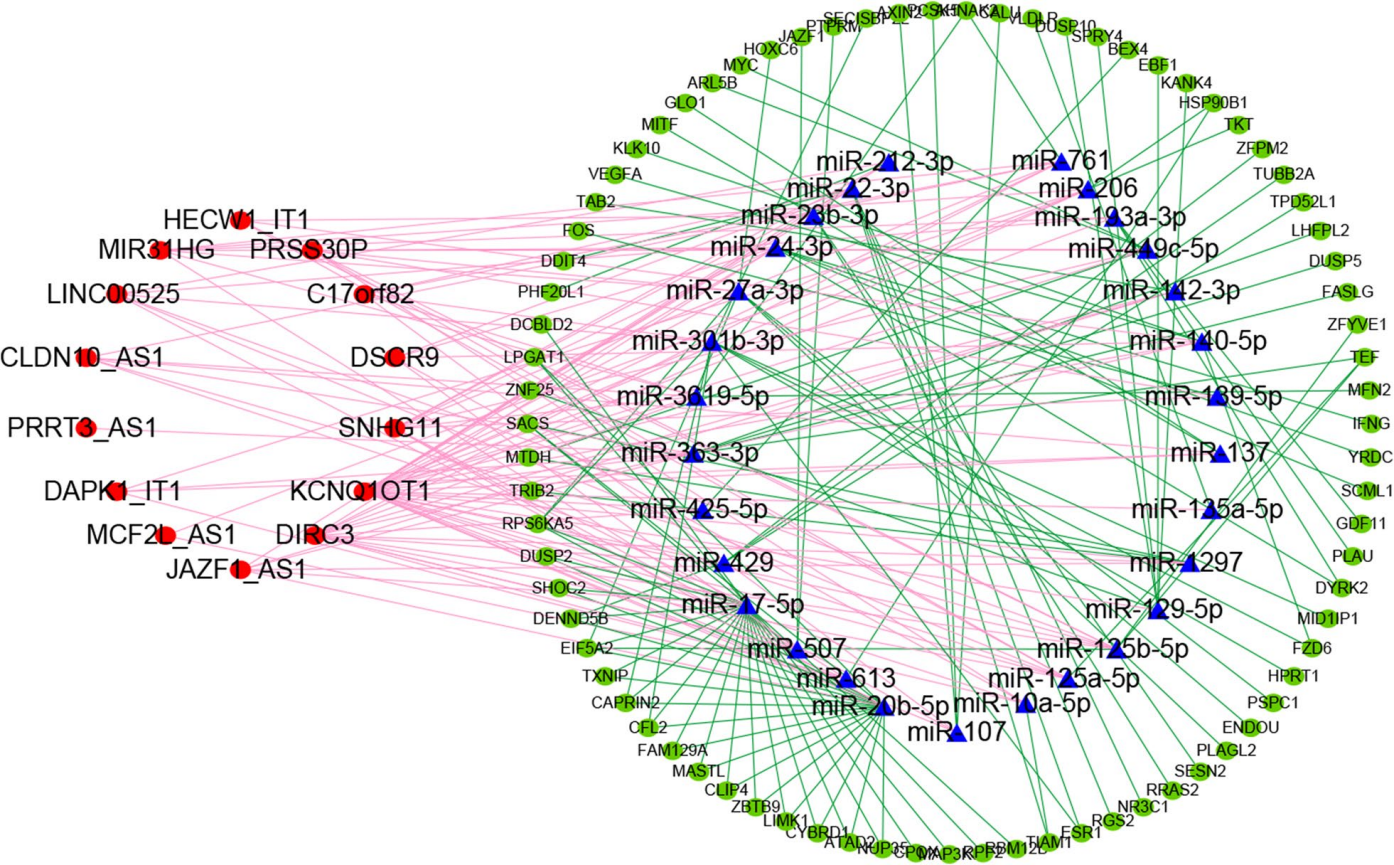
Competitive endogenous RNA network chart

The miRNA- targeted mRNAs that could be searched in three above databases were defined as the miRNA-targeted mRNAs. Third, these miRNA-targeted mRNAs were intersected with previous prognostic mRNAs for development of ceRNA network. Finally, the ceRNA network, consisting of 14 lncRNAs, 29 miRNAs, and 79 mRNAs, were constructed for CRC. The ceRNA network was visualized in Fig. 1 by Cytoscape v3.6.1. This ceRNA network depicted potential regulatory relations among lncRNAs, miRNAs, and mRNAs, and was helpful to understand the potential mechanisms of tumor prognosis.

### Functional enrichment analyses

**Fig. 2:**
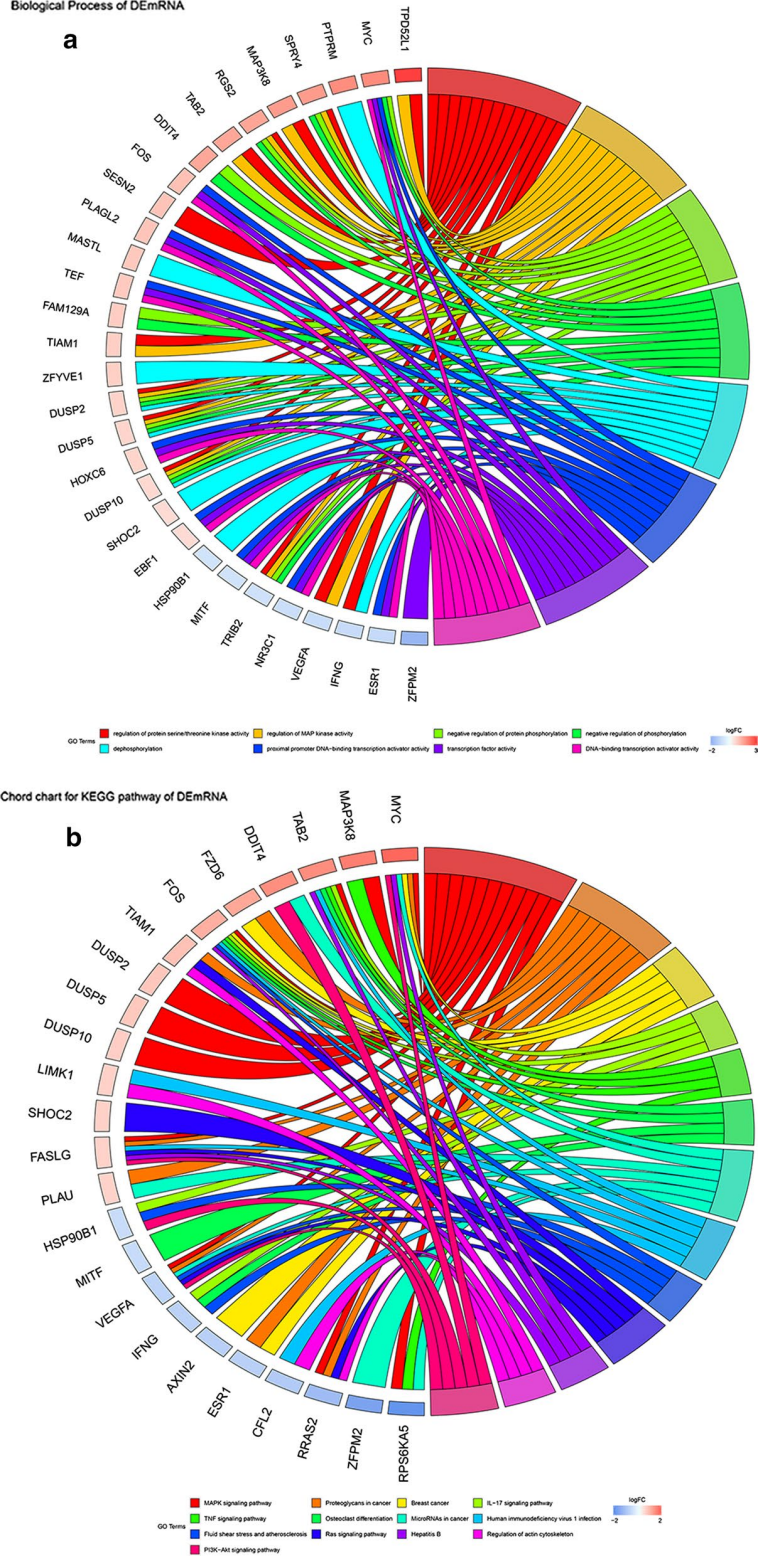
Chord chart of prognostic mRNAs: **a** GO terms; **b** KEGG pathways

**Fig. 3:**
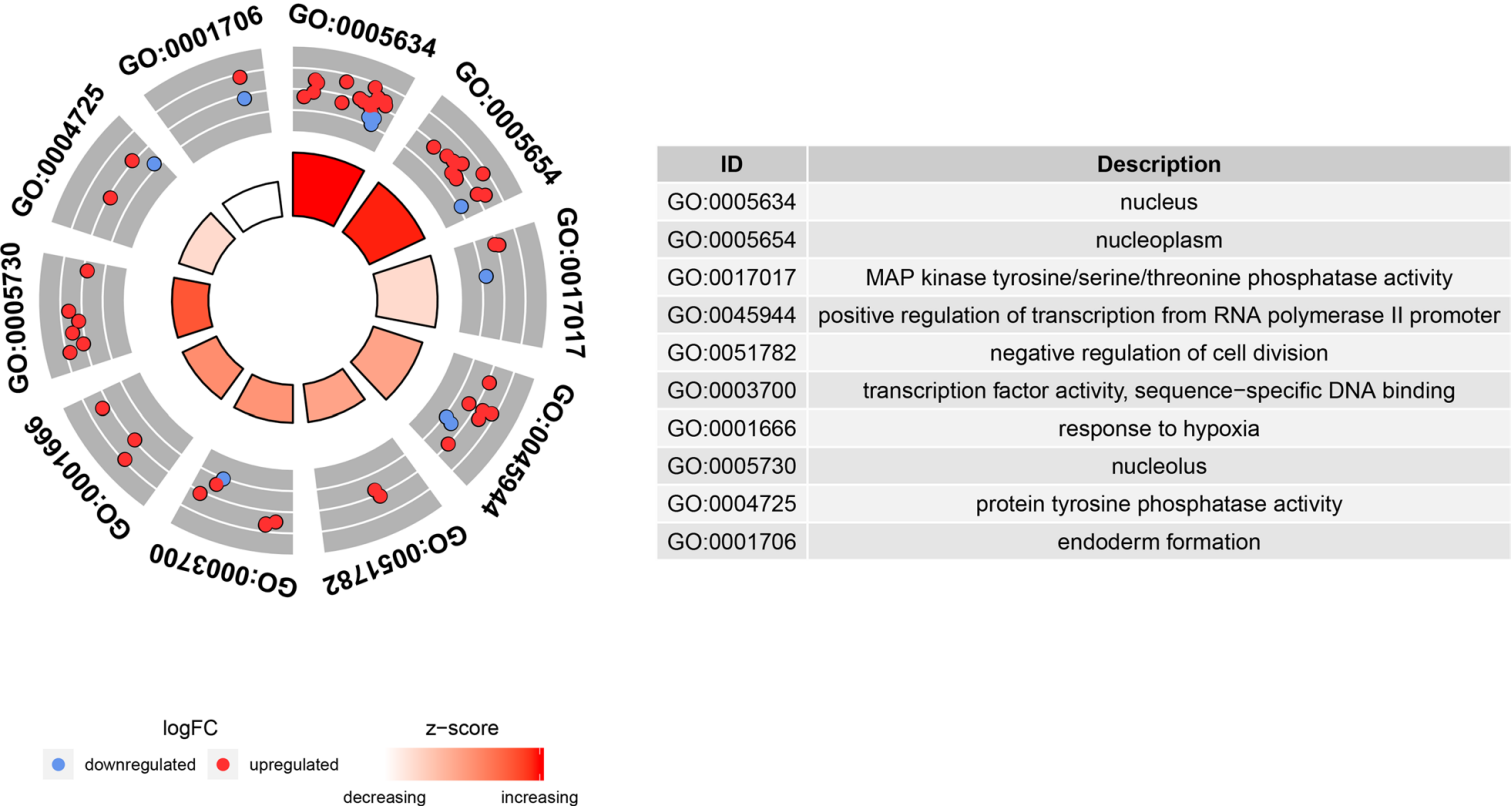
Circular chart of prognostic mRNAs

Functional enrichment analyses were performed based on previous prognostic mRNAs in ceRNA network and identified 26 enriched Gene Ontology (GO) terms. The top 15 enriched GO terms were shown in Fig. 2a. The prognostic mRNAs were mainly enriched in transcription factor activity, RNA polymerase II proximal promoter sequence-specific DNA binding, regulation of MAP kinase activity, regulation of protein serine/threonine kinase activity, inactivation of MAPK activity, MAP kinase tyrosine/serine, MAP kinase phosphatase activity, protein tyrosine/serine, E-box binding, protein tyrosine phosphatase activity, and negative regulation of MAP kinase activity. The top KEGG pathways (Fig. 2b) were mainly enriched in MAPK signaling pathways, Proteoglycans in cancer, Breast cancer, IL-17 signaling pathway, TNF signaling pathway, Osteaclast differentiation, MicorRNAs in cancer, Human immunodeficiency virus 1 infection, Fluid shear stress and atherosclesosis, Ras signaling pathway, Hepatitis B, Regulation of action cytoskeleton, PI3K-Akt signaling pathway. These biological functions were helpful to understand the roles of these genes in tumor prognosis. The further circular visualization chart was presented in Fig. 3.

### Construction of predictive model

**Fig. 4:**
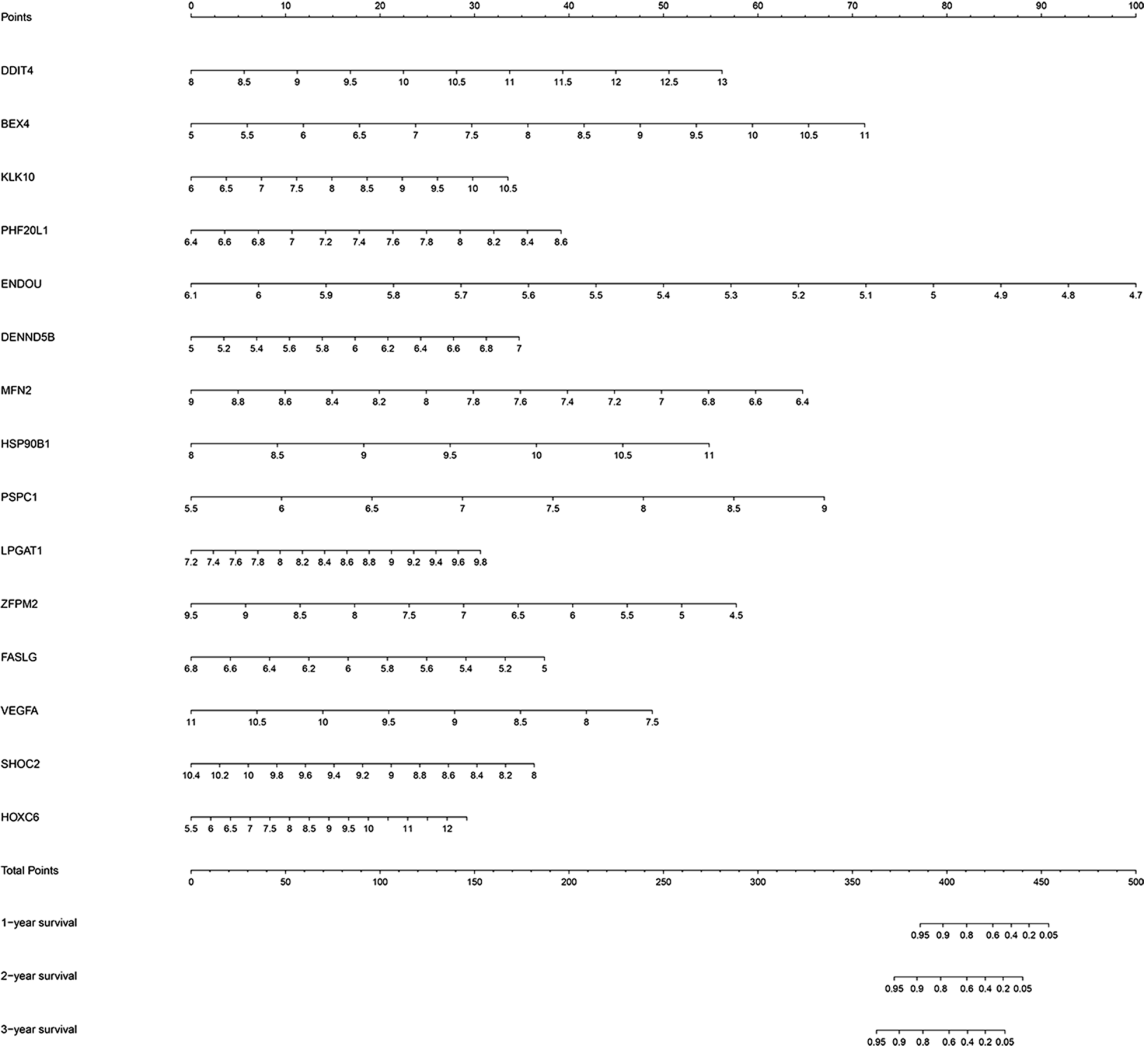
Prognostic nomogram for overall survival in colorectal cancer

Multivariate Cox regression analyses were used to screen independent risk factors for tumor prognosis. Fifteen mRNAs were used to develop a predictive model for CRC (Table 2). The formula for predictive model was as follows: The risk score = (− 4.273 * ENDOU) + (− 1.489 * MFN2) + (− 1.243 * FASLG) + (− 0.904 * SHOC2) + (− 0.834 * VEGFA) + (− 0.690 * ZFPM2) + (0.249 * HOXC6) + (0.446 * KLK10) + (0.672 * DDIT4) + (0.705 * LPGAT1) + (0.711 * BEX4) + (1.038 * DENND5B) + (1.065 * PHF20L1) + (1.093 * HSP90B1) + (1.146 * PSPC1). The prognostic nomogram chart was shown in Fig. 4.

**Table 2:** Model information of prognostic mRNAs

Gene	Univariate analysis			Multivariate analysis			
	HR	95% CI	*P*-value	Coefficient	HR	95% CI	*P*-value
ENDOU	0.642	0.425–0.972	0.036	− 4.273	0.014	0.004–0.047	< 0.001
MFN2	0.632	0.417–0.957	0.030	− 1.489	0.226	0.125–0.408	< 0.001
FASLG	0.560	0.368–0.853	0.007	− 1.243	0.288	0.098–0.846	0.024
SHOC2	1.544	1.023–2.333	0.039	− 0.904	0.405	0.179–0.917	0.030
VEGFA	1.569	1.037–2.373	0.033	− 0.834	0.434	0.226–0.833	0.012
ZFPM2	1.805	1.183–2.753	0.006	− 0.690	0.501	0.363–0.693	< 0.001
HOXC6	1.567	1.037–2.368	0.033	0.249	1.283	1.064–1.547	0.009
KLK10	1.593	1.053–2.409	0.028	0.446	1.562	1.161–2.100	0.003
DDIT4	1.710	1.126–2.596	0.012	0.672	1.959	1.319–2.908	0.001
LPGAT1	1.570	1.039–2.371	0.032	0.705	2.023	1.199–3.415	0.008
BEX4	1.795	1.185–2.721	0.006	0.711	2.036	1.521–2.725	< 0.001
DENND5B	1.527	1.010–2.308	0.045	1.038	2.824	1.155–6.909	0.023
PHF20L1	1.562	1.028–2.373	0.037	1.065	2.901	1.162–7.241	0.022
HSP90B1	1.959	1.291–2.972	0.002	1.093	2.982	1.561–5.696	0.001
PSPC1	1.863	1.228–2.825	0.003	1.146	3.144	1.682–5.877	< 0.001

*HR* hazard ratio, *CI* confidence interval

### Survival curve analyses

Survival curve analyses were performed to explore the survival influences of included mRNAs (Additional file 1: Figure S2). Kaplan–Meier curves indicated that patients with high expression had significantly poor overall survival than that with low expression for these 15 mRNAs (*P *< 0.05). Comparison of Kaplan–Meier curves supported that these 15 genes were associated with overall survival in CRC patients.

### Predictive performance in model dataset

**Fig. 5:**
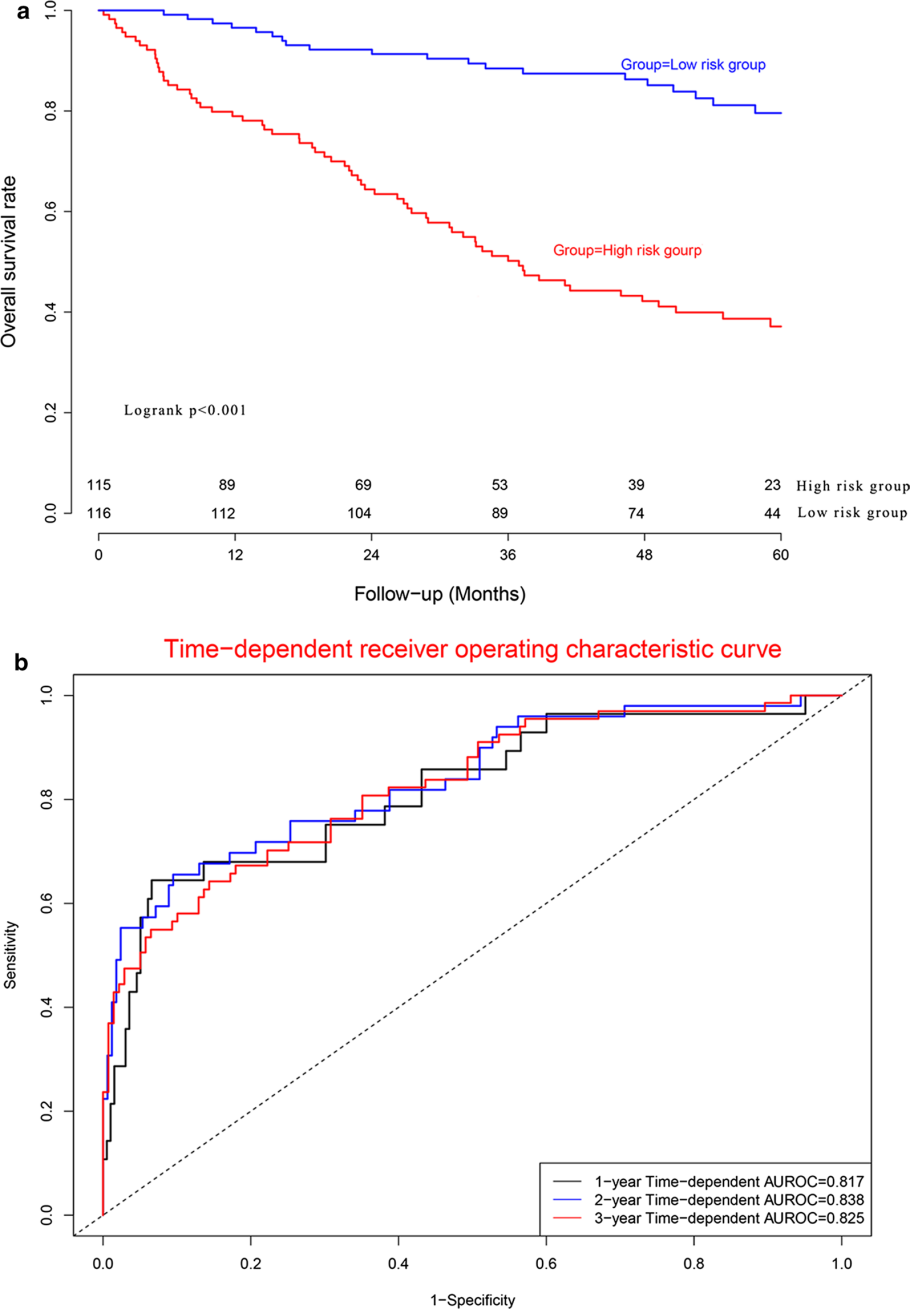
Clinical performance in model cohort: **a** survival curve plot; **b** time-dependent receiver operating characteristic curve plot

**Fig. 6:**
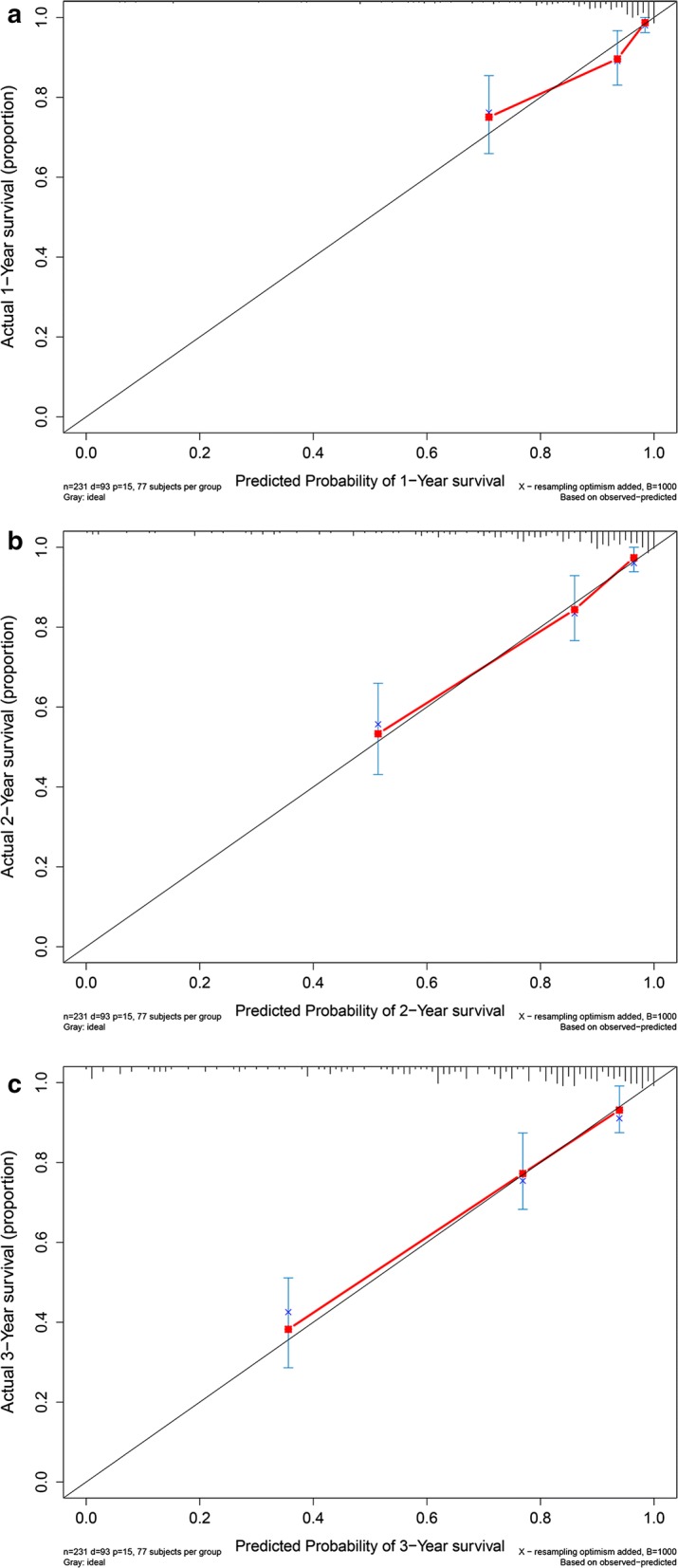
Calibration curves for model cohort. **a** Calibration curve for 1-year survival; **b** calibration curve for 2-year survival; **c** calibration curve for 3-year survival

CRC patients were divided into high risk subgroup and low risk subgroup according to median risk score. Figure 5a demonstrated that there was significant difference between two subgroups for OS (*P *< 0.001). Concordance indexes were 0.817, 0.838, and 0.825 respectively for 1-year, 2-year, and 3-year OS (Fig. 5b). Calibration curves for OS were presented in Fig. 6, indicating a good agreement between predicted mortality and actual mortality for 1-year, 2-year, and 3-year OS.

### Predictive performance in validation dataset

**Fig. 7:**
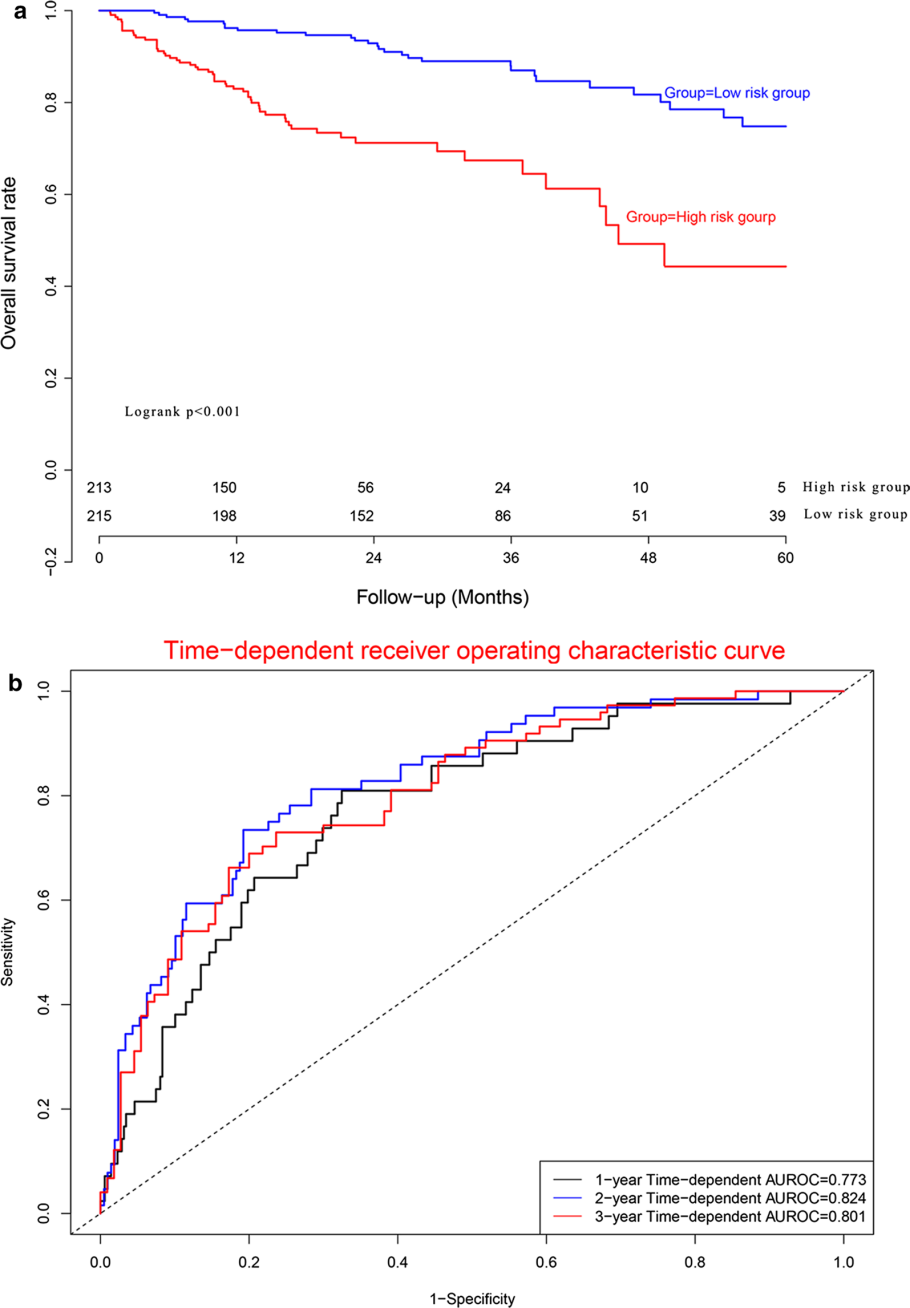
Clinical performance in validation cohort: **a** survival curve plot; **b** time-dependent receiver operating characteristic curve plot

**Fig. 8:**
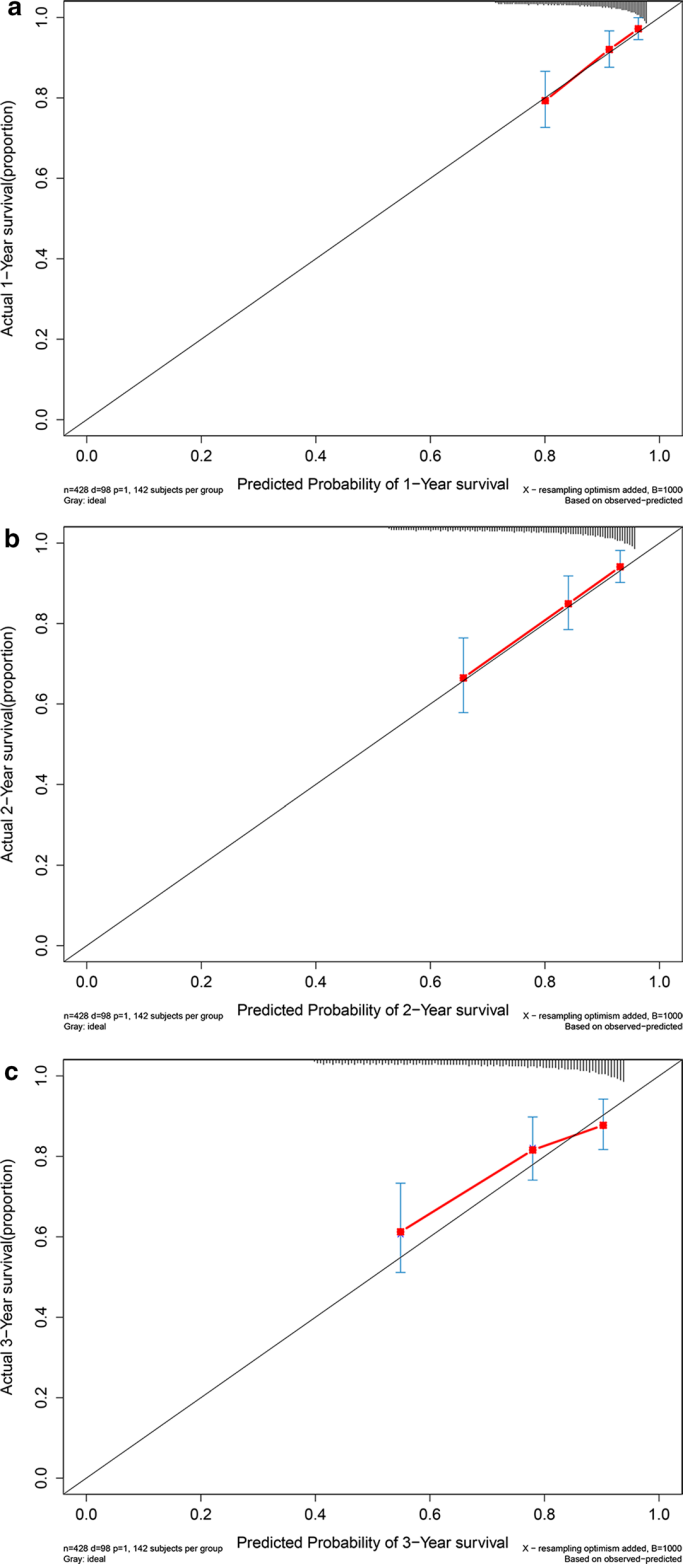
Calibration curves for validation cohort. **a** Calibration curve for 1-year survival; **b** calibration curve for 2-year survival; **c** calibration curve for 3-year survival

Kaplan–Meier plot (Fig. 7a) demonstrated that OS in high risk subgroup was significantly worse than that in low risk subgroup (*P *< 0.05). Concordance indexes were 0.773, 0.824, and 0.801 for 1-year, 2-year, and 3-year OS (Fig. 7b). Calibration curves for OS were depicted in Fig. 8, demonstrating that the predicted mortality was in good agreement with the actual mortality.

### Independence assessment of predictive model

In model cohort, prognostic signature and pathological stage were independent influence factors for OS (Table 3). In validation cohort, prognostic signature, pathological stage, and age were identified as independent influence factors for OS. Decision curves and clinical impact curve for OS were presented in Additional file 1: Figure S3, suggesting that the clinical efficacy of the current prognostic model was superior to pathological stage and age.

**Table 3:** Independence assessment of prognostic model

	Univariate analysis			Multivariate analysis			
	HR	95% CI	*P*-value	coefficient	HR	95% CI	*P*-value
Model group							
Gender (male/female)	0.883	0.586–1.33	0.551	− 0.247	0.781	0.508–1.202	0.261
Age (high/low)	0.591	0.381–0.917	0.018	0.161	1.175	0.765–1.804	0.463
AJCC stage (IV, III/II, I)	0.323	0.21–0.498	< 0.001	1.196	3.306	1.979–5.522	< 0.001
Prognostic model (high/low)	6.48	2.037–20.61	< 0.001	1.499	4.476	2.706–7.403	< 0.001
Validation group							
Gender (male/female)	0.993	0.66–1.496	0.975	− 0.089	0.915	0.608–1.379	0.672
Age (high/low)	0.942	0.627–1.415	0.773	0.712	2.037	1.330–3.120	< 0.001
AJCC stage (IV, III/II, I)	0.270	0.163–0.448	< 0.001	1.206	3.341	2.185–5.107	< 0.001
Prognostic model (high/low)	0.210	0.129–0.342	< 0.001	− 1.228	0.293	0.189–0.453	< 0.001

*AJCC* The American Joint Committee on Cancer, *HR* hazard ratio, *CI* confidence interval

### Smart Cancer Survival Predictive System

**Fig. 9:**
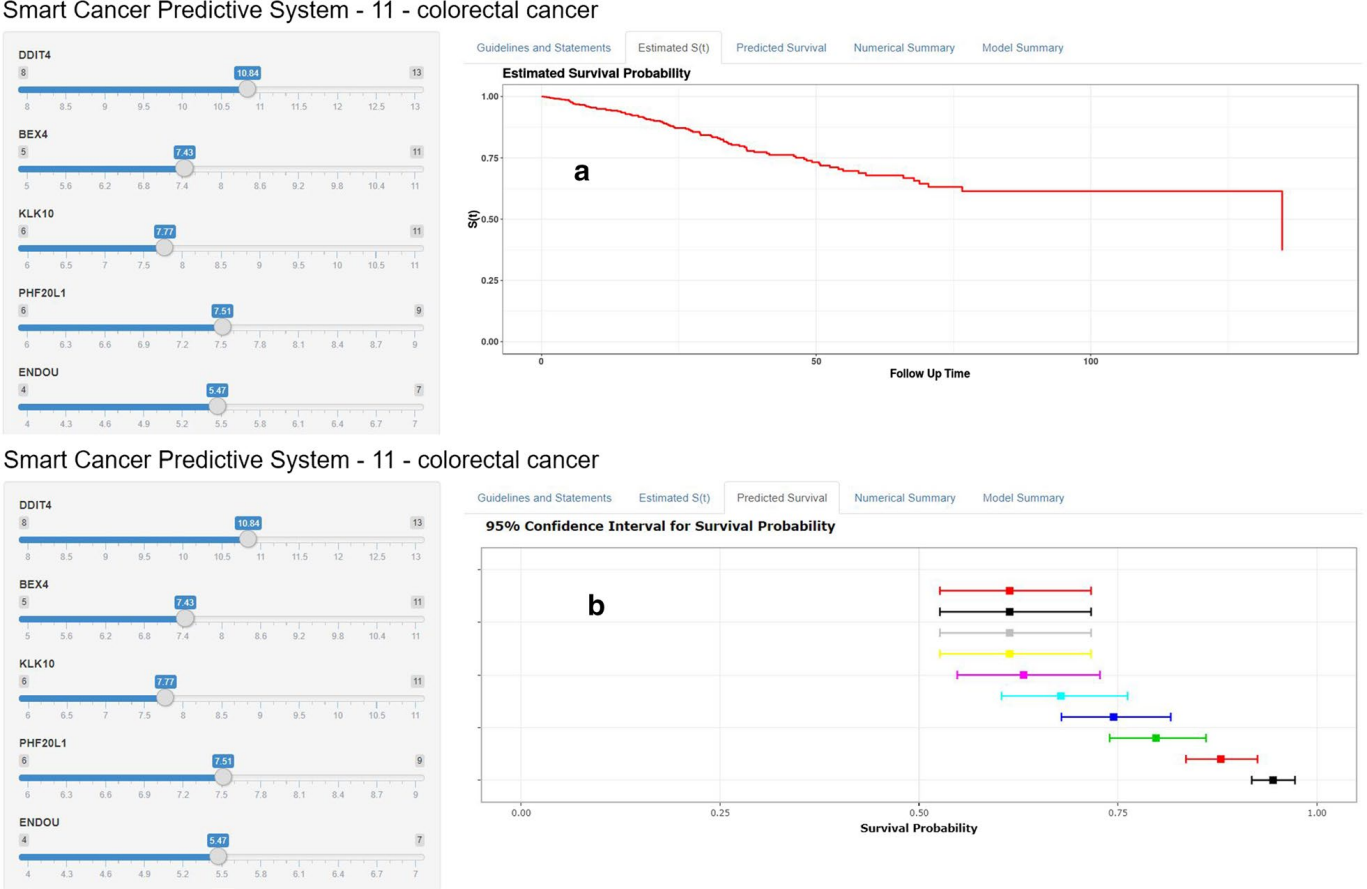
Home page of Smart Cancer Survival Predictive System. **a** Survival curves display page; **b** quantitative display page

We developed a precision medicine predictive tool named Smart Cancer Survival Predictive System, providing a novel convenient on-line calculator for prediction of OS. Smart Cancer Survival Predictive System (Fig. 9) is available at: https://zhangzhiqiao6.shinyapps.io/Smart_cancer_predictive_system_11_CRC_B1003/. Smart Cancer Survival Predictive System provided three individual mortality risk curves predicted by Random survival forest algorithm, Multitask logistic regression algorithm, and Cox survival regression algorithm according to the calculation formula in ogininal articles.

### Gene Survival Analysis Screen System

**Fig. 10:**
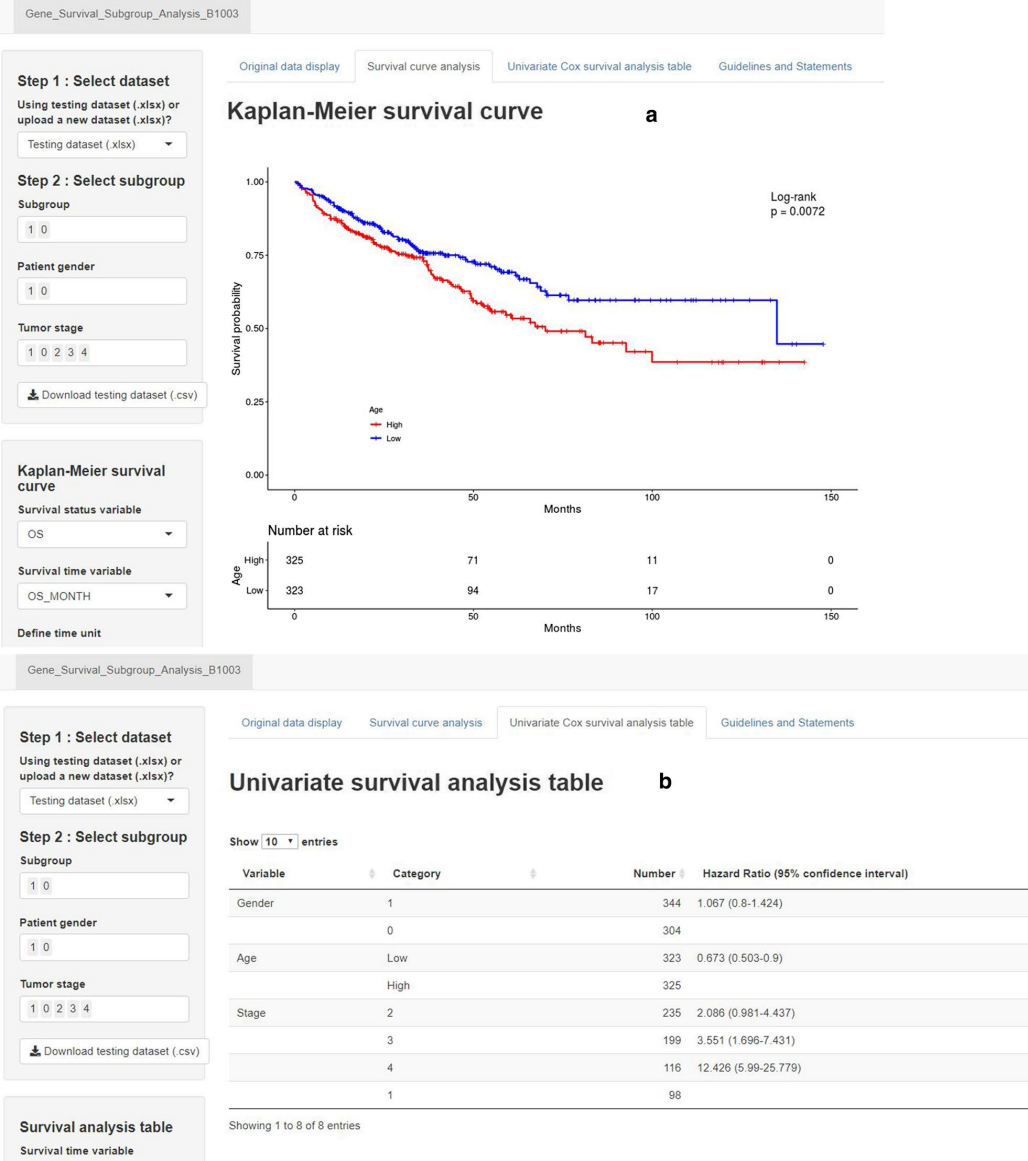
Home page of Gene Survival Analysis Screen System. **a** Survival curves display page; **b** Cox survival analysis display page

To further explore survival curves of previous prognostic genes in different gender and pathological stage subgroups, we developed a new online program named Gene Survival Analysis Screen System (Fig. 10). Gene Survival Analysis Screen System is available at: https://zhangzhiqiao5.shinyapps.io/Gene_Survival_Subgroup_Analysis_B1003/. Gene Survival Analysis Screen System provided seven tumor datasets for exploration research and allowed users to select tumor type, gender, and stage, which were the important influence factors to the prognosis.

### Clinical application in other tumors

**Fig. 11:**
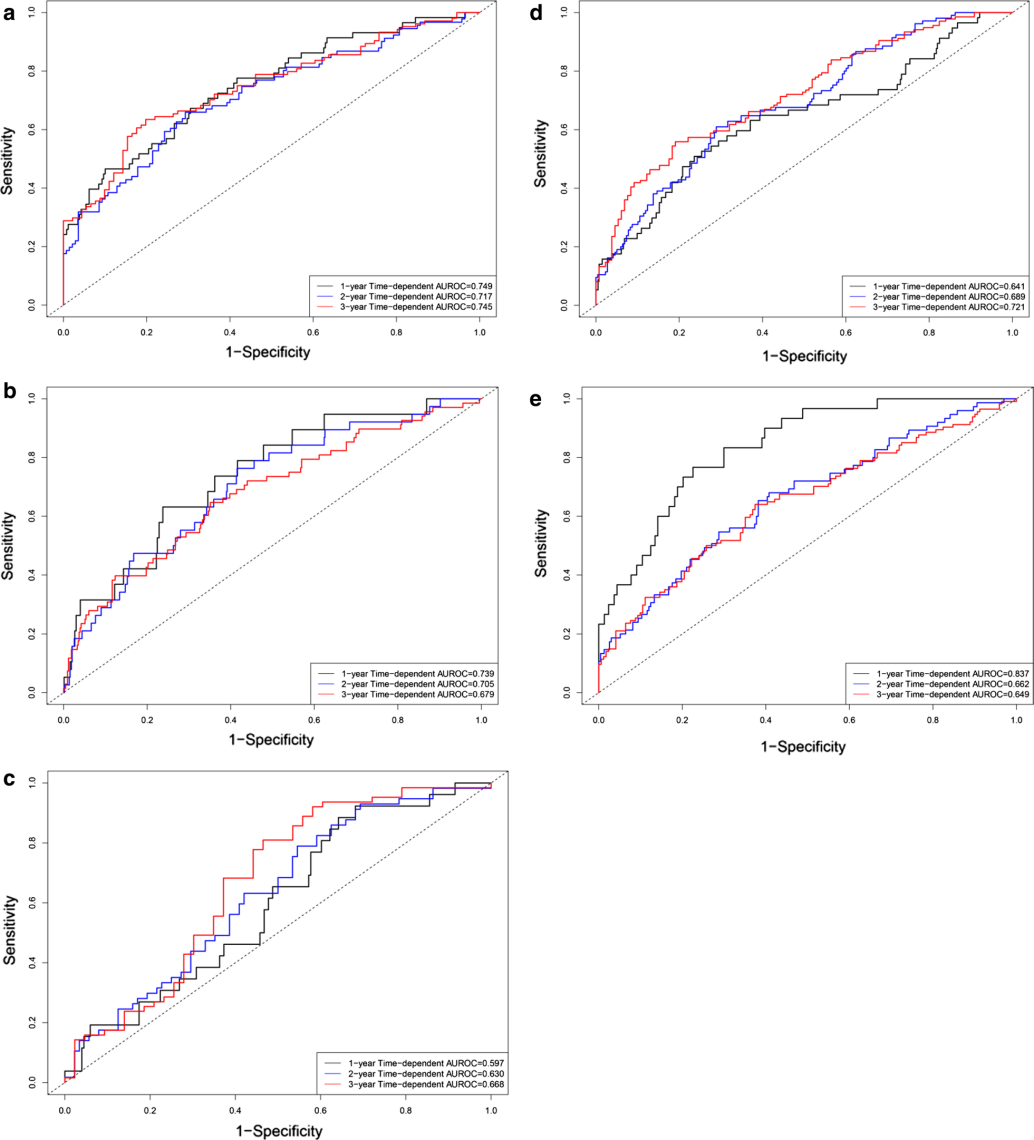
Time-dependent receiver operating characteristic curve plots. **a** Hepatocellular carcinoma; **b** breast cancer; **c** gastric cancer; **d** lung cancer; **e** ovarian cancer

The current study download five different tumor datasets from TCGA database as external validation datasets to explore the clinical application of the current prognostic model. Figure 11 displayed the diagnostic accuracy of the current prognostic model in five tumors, including hepatocellular carcinoma (n = 348), breast cancer (n = 1030), gastric cancer (n = 265), lung cancer (n = 494), and ovarian cancer (n = 370).

### Predictive performance in five malignant solid tumors

**Fig. 12:**
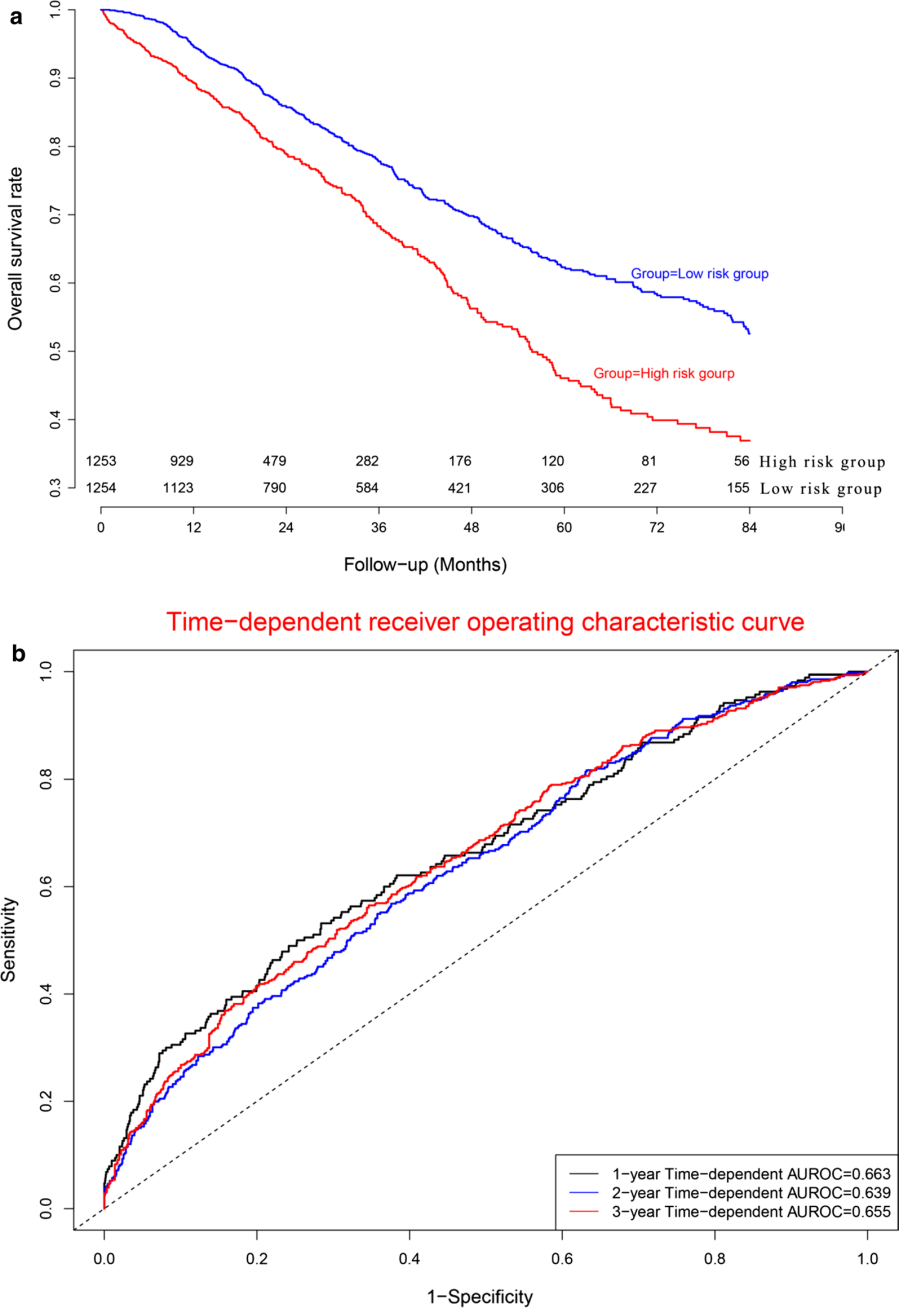
Clinical performance in five malignant solid tumor datasets: **a** survival curves for high risk group and low risk group. **b** Time-dependent receiver operating characteristic curves

**Fig. 13:**
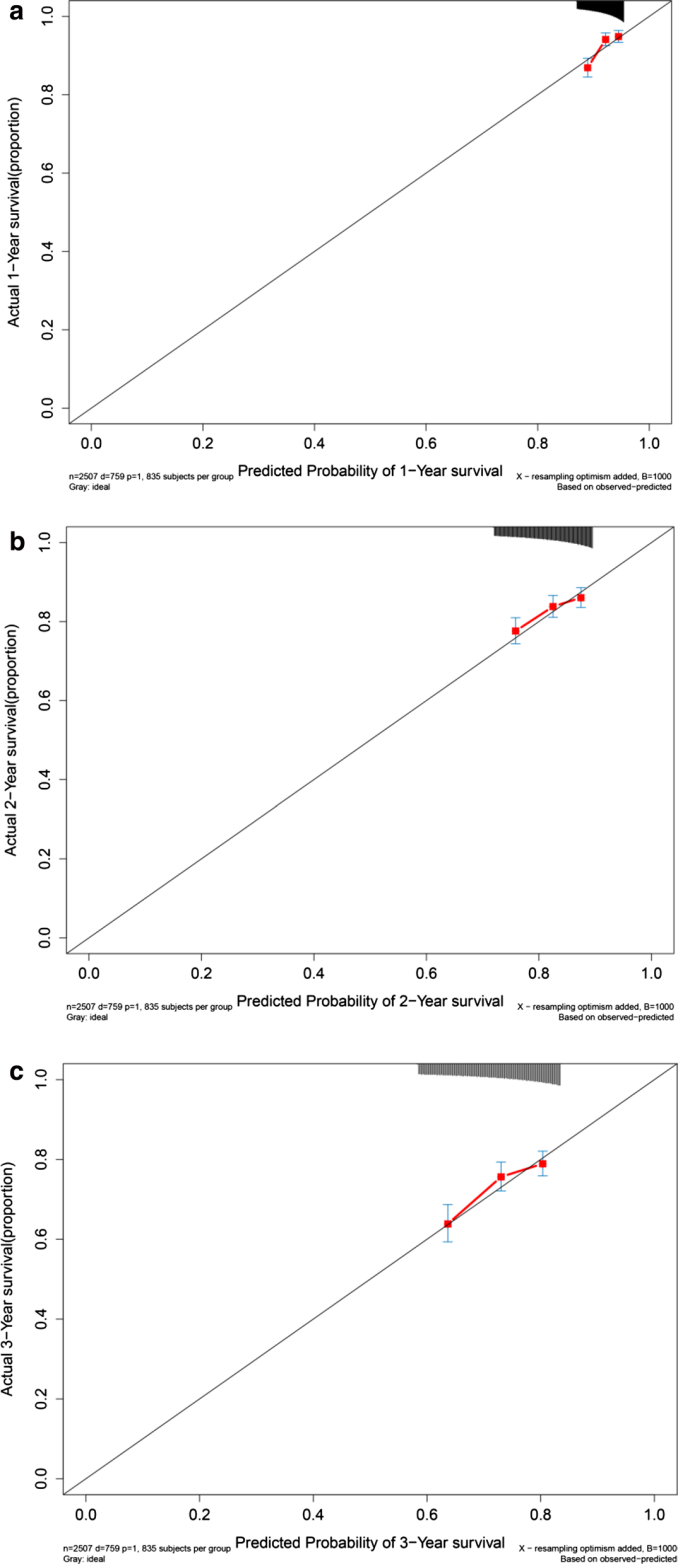
Calibration curves in five malignant solid tumor datasets: **a** calibration curve for 1-year survival; **b** calibration curve for 2-year survival; **c** calibration curve for 3-year survival

To further explore the predictive performance of the current prognostic model, we externally validated the current prognostic model in a super merge dataset (n = 2507), which including five malignant solid tumors (hepatocellular carcinoma, breast cancer, gastric cancer, lung cancer, and ovarian cancer). Kaplan–Meier plot (Fig. 12a) demonstrated that OS in high risk subgroup was significantly worse than that in low risk subgroup (*P *< 0.05). Concordance indexes were 0.663, 0.639, and 0.655 for 1-year, 2-year, and 3-year OS (Fig. 12b). Calibration curves for OS were depicted in Fig. 13, demonstrating that the predicted mortality was in good agreement with the actual mortality.

## Discussion

The current research constructed a prognosis-related ceRNA network for CRC. A prognostic nomogram was developed for OS based on prognostic mRNAs in ceRNA network. The current prognostic model could discriminate high risk patients from low risk patients for different time points. Meanwhile, two novel precision medicine predictive tools were developed to provide convenient on-line calculation for prediction of OS in CRC patients. Smart Cancer Survival Predictive System provided three individual mortality risk curves predicted by Random survival forest algorithm, Multitask logistic regression algorithm, and Cox survival regression. Gene Survival Analysis Screen System provided survival curve comparison and multivariable survival analysis results. Smart Cancer Survival Predictive System and Gene Survival Analysis Screen System could provide precious individualized mortality risk prediction for CRC patients.

The current study screened differentially expressed RNAs between CRC tissues and normal tissues and then constructed a prognosis-related ceRNA regulatory network for CRC. Based on mRNAs in ceRNA regulatory network, the current study further identified independent prognostic biomarkers for overall survival in CRC patients. Through ceRNA regulatory network, the regulatory relationship among lncRNA-miRNA-mRNA was depicted, providing post-transcriptional biological regulatory pathway information for CRC. GO term and KEGG pathway analyses were helpful to further understand the biological function and molecular regulatory pathway of prognostic mRNAs in ceRNA network. The fifteen prognostic mRNAs were identified as independent prognostic biomarkers for overall survival in CRC patients by multivariable Cox regression. The ceRNA regulatory network and functional enrichment analyses provided potential biological prognostic indicators and therapeutic targets for future researches.

Mitofusin 2 (MFN2) promotes cell proliferation and invasiveness in gastric cancer [30]. Lin et al. reported that miRNA-30d can regulate cancer angiogenesis and cancer proliferation through MYPT1/c-JUN/VEGFA pathway [31]. Liu et al. reported that ZFPM2 Antisense RNA 1 (ZFPM2-AS1) promotes tumor proliferation and inhibits apoptosis through regulating miR-137 in renal cell cancer [32]. Li et al. reported that Homeobox C6 (HOXC6) promotes invasion via EMT pathway in hepatocellular carcinoma [33]. Petraki et al. reported that Kallikrein Related Peptidase 10 (KLK10) expression is associated with overall survival in CRC [34]. Xu et al. reported that MiR-199b-5p can promote tumor proliferation through regulating KLK10 in cervical cancer [35]. Liu et al. reported that Heat Shock Protein 90 Beta Family Member 1 (HSP90B1) is significantly related with worse overall survival in lung cancer [36]. Cawthorn et al. reported that up-regulated HSP90B1 is related with distant metastasis [37]. Kessler et al. reported that Paraspeckle Component 1 (PSPC1) is significantly related with poor prognosis in hepatocellular carcinoma [38]. The results in current study further supported the credibility of these findings above in previous researches.

High quality samples and standardized RNA extraction technology are helpful to improve the quality and reliability of research data. According to the Standard Operating Procedure suggested by TCGA database, for gene sequencing sample, conventional methods of fixation and heating of biopsies may lead to inactivation of antigens and genetic material in tissues, therefore frozen sections was recommended in TCGA database. Samples should be snap-frozen and stored in cryovials in liquid nitrogen vapor and should not allow tissues thaw until sectioned. The mirVana isolation technology uses organic extraction and then fixes RNA on glass fiber filter to purify total RNA, so it can prepare high-purity and high-quality small RNA molecules within 2 h. RNA analytes should not undergo multiple freeze–thaw cycles to maintain biological activity. RNA is easily degraded by ribonuclease, so special methods should be taken to prevent RNA degradation. All reagents must be made of ribonuclease free materials and all products and disposable materials used must be free of ribonuclease. In order to prevent contamination by ribonuclease carried by the skin, gloves must be worn before handling biological samples. RNA analytes should be placed on wet ice and RNA quantification should be performed before freezing.

Advantages of the current study: Firstly, our study team developed a precision medicine tool named Smart Cancer Survival Predictive System, providing full-time individual mortality risk prediction with visual illustration and numerical presentation. Secondly, a novel precision medicine tool named Gene Survival Analysis Screen System was developed to explore the associations between prognostic genes (including clinical parameters) and overall survival. Users are free to choose the appropriate subgroup, gender, and stage. Meanwhile, users can upload their own dataset to explore and validate the research result.

Shortcomings of the current research: first, detection platforms were different between model dataset and validation dataset. The influence of different detection platforms on gene expression read counts should be taken into account while evaluating clinical application of the current prognostic model. Second, several important prognostic factors were not included in the current study, including surgical, radiotherapy and chemotherapy regimens. Third, the prognostic model was developed and validated by using datasets downloaded from public databases without research team’s study cohort. External applicability of prognostic model needs to be validated by study cohorts from different population.

## Conclusions

In conclusion, the current study provided deeper understanding of prognosis-related ceRNA regulatory network for CRC. Two precision medicine predictive tools named Smart Cancer Survival Predictive System and Gene Survival Analysis Screen System were constructed for CRC. These two precision medicine predictive tools can provide individual prognostic information before surgery and improve the individualized treatment decision-making.

## Supplementary information

**Additional file 1: Figure S1.** Volcano plot: (A) Volcano plot of lncRNAs; (B) Volcano plot of miRNAs; (C) Volcano plot of mRNAs. **Figure S2.** Survival curves of prognostic genes. **Figure S3.** Decision curves and clinical impact curves.

## Data Availability

The study data is available at: https://zhangzhiqiao5.shinyapps.io/Gene_Survival_Subgroup_Analysis_B1003/.

## Abbreviations

CRC: colorectal cancer
TCGA: The Cancer Genome Atlas
GEO: The Gene Expression Omnibus
ROC: receiver operating characteristic
OS: overall survival
lncRNA: long non-coding RNA
miRNA: microRNAs
HR: hazard ratio
CI: confidence interval
AJCC: The American Joint Committee on Cancer
SD: standard deviation
DCA: decision curve analysis
ceRNA: competitive endogenous RNA
