# Supplementary material for: Two precision medicine predictive tools for six malignant solid tumors: from gene-based research to clinical application
Source: J Transl Med. 2019 Dec 3;17:405. doi: 10.1186/s12967-019-02151-8 (PMC6891961; doi:10.1186/s12967-019-02151-8)

Additional file 1: Figure S1. Volcano plot: (A) Volcano plot of lncRNAs; (B) Volcano plot of miRNAs; (C) Volcano plot of mRNAs.


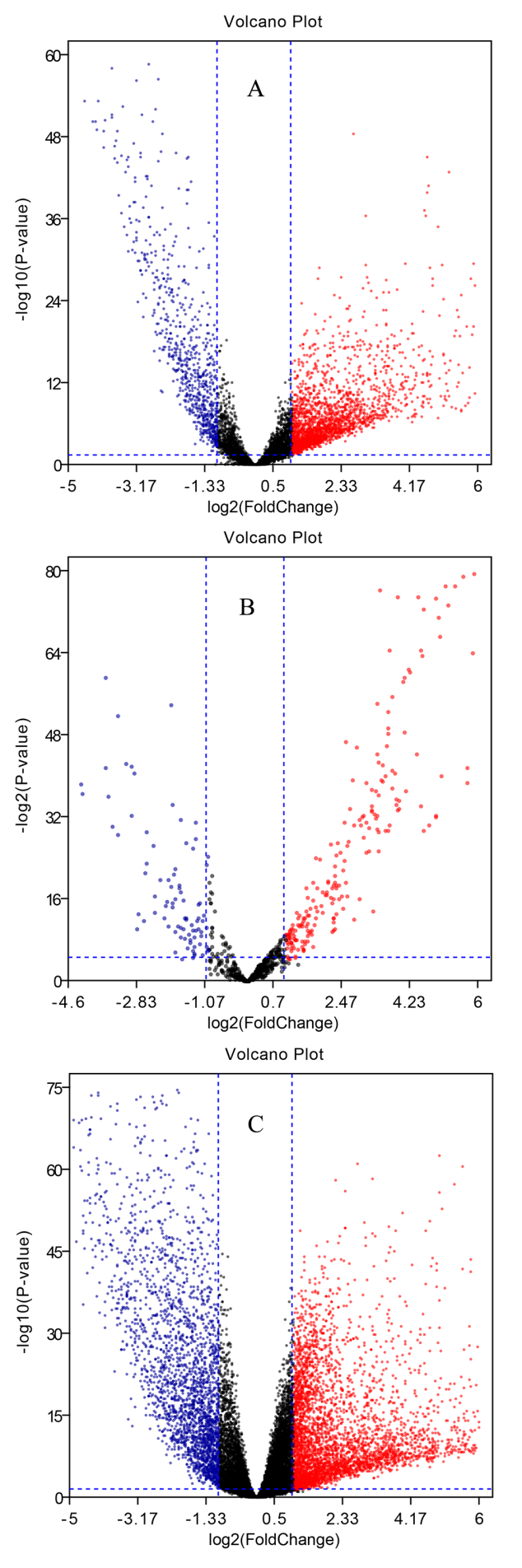


Additional file 1: Figure S2. Survival curves of prognostic genes.


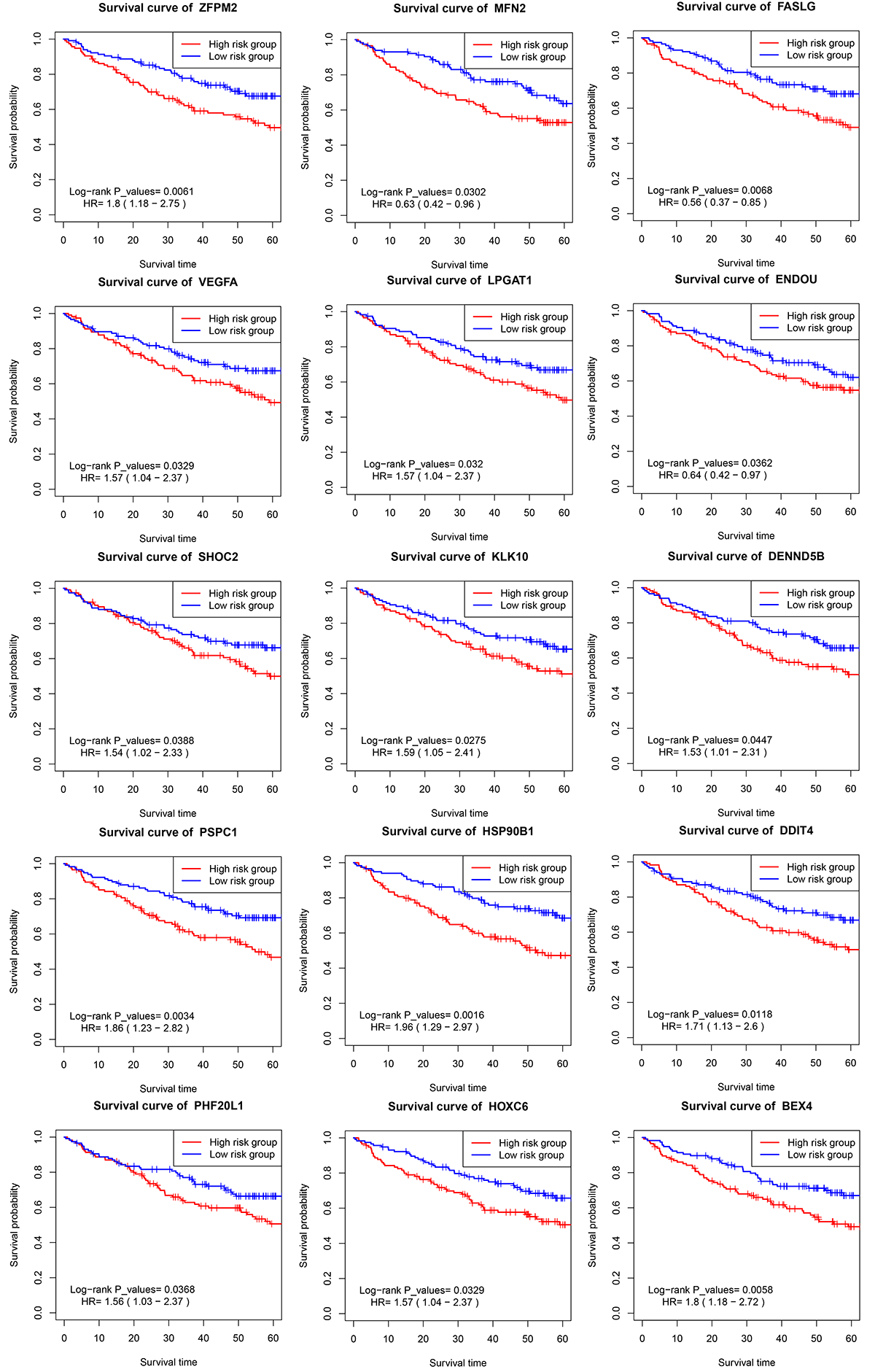


Additional file 1: Figure S3. Decision curves and clinical impact curves: decision curve analysis for 1-year disease free survival (A); decision curve analysis for 2-year disease free survival (B); decision curve analysis for 3-year disease free survival (C); clinical impact curve (D). The y-axis represented the net benefit. The red solid line represented the prognostic signature. The grey solid line represented the net benefit of treating all patients within 1-, 2-, and 3year. The black solid line represented the net benefit of treating no patients within 1-, 2-, and 3year.


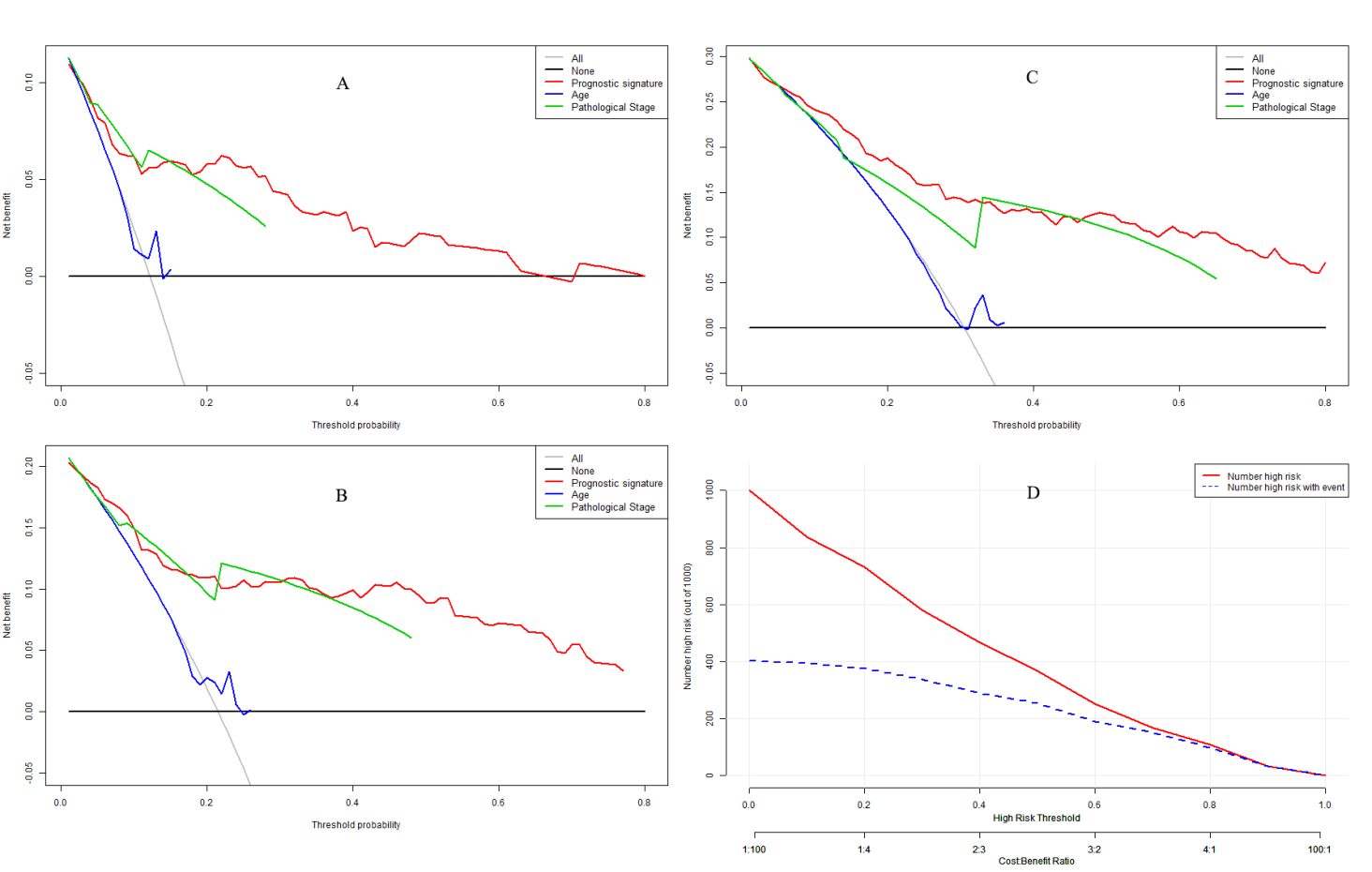

Supplement: Supplementary file 1 — Additional file 1: Figure S1. Volcano plot: (A) Volcano plot of lncRNAs; (B) Volcano plot of miRNAs; (C) Volcano plot of mRNAs. Figure S2. Survival curves of prognostic genes. Figure S3. Decision curves and clinical impact curves. [file 12967_2019_2151_MOESM1_ESM.docx]
